# Molecular crosstalk between circadian clock and cancer and therapeutic implications

**DOI:** 10.3389/fnut.2023.1143001

**Published:** 2023-03-02

**Authors:** Meng Qu

**Affiliations:** International Institutes of Medicine, The Fourth Affiliated Hospital of Zhejiang University School of Medicine, Yiwu, Zhejiang, China

**Keywords:** circadian rhythm, cancer, clock-targeting, chronomedicine, chronotherapy

## Abstract

The circadian clock governs activity of many physiological processes, thereby playing a pivotal role in human health. Circadian disruption is closely associated with cancer development; in particular, recent discoveries have provided strong evidence supporting specific functions of different molecular clock components in either promoting or inhibiting tumorigenesis. This narrative review aims to summarize the existing data on molecular connections between the clock and cancer. These results along with future efforts pave the road to targeting the circadian clock as a novel pathway for therapeutic intervention. Given the implications of chrono-nutrition interventions such as time-restricted feeding in extending lifespan, chrono-nutrition may have preventive and therapeutic applications for individuals with and at-risk of age-related diseases including cancer.

## Introduction

Evolved by most living creatures, the circadian clock allows organisms to organize their behavior and physiology to anticipate diurnal environmental changes. The mammalian circadian machinery is centrally controlled by core clock genes which form two interlocked transcriptional feedback loops. The BMAL1::CLOCK (brain and muscle ARNT-like 1 and CLOCK) heterodimer activates transcription of the clock repressors PER1/2/3 and CRY1/2, which subsequently form a heterodimer, translocate into the nucleus, and inhibit transcriptional activity of BMAL1::CLOCK. In the other loop, RORs and REV-ERBs, which are also targets of BMAL1::CLOCK, feedback to collaboratively regulate BMAL1::CLOCK by stimulating or repressing *Bmal1* transcription, respectively (1). Up to 80% of the protein-coding genes exhibit circadian transcription somewhere in the body of primates, highlighting the molecular clock as a crucial mechanism monitoring a broad range of physiological functions, including sleep, feeding, hormone secretion, metabolism, and immune responses (2, 3). Environmental or genetic disruption of circadian homeostasis exacerbates the development of clinically relevant disorders, such as sleep disorders, obesity, diabetes, and cancers, prompting pharmacological manipulation of the clock for novel treatments (4).

The normal circadian rhythms have long been acknowledged as a tumor suppressor, given that epidemiologic studies have suggested night shift work being a carcinogenic factor, particularly for breast cancer (5, 6). Systemic studies also revealed circadian clock genes are frequently dysregulated or mutated across many human cancer types (7–9). Furthermore, the disruption of circadian rhythms is closely associated with cancer incidence, stage, and survival rate (7, 10, 11). While recent discoveries in different cancer systems have confirmed the significant roles of the circadian machinery in cancer biology, studies conducted in specific cancer systems have provided consistent evidence showing that certain central clock molecules may play oncogenic roles (10). Devising strategies for clock-based cancer therapeutics therefore calls for in-depth mechanistic studies to guide cancer-specific treatments for optimal efficacy and safety. This article will discuss the broad spectrum of molecular functions of the circadian clock in driving cancer progression.

## Circadian control of cell cycle progression and DNA damage response

**FIGURE 1:**
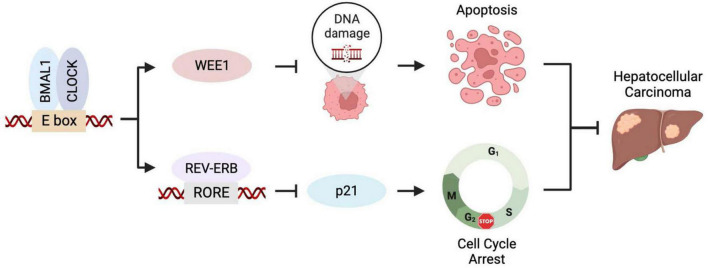
BMAL1::CLOCK promotes HCC growth by controlling cell cycle regulators. BMAL1::CLOCK directly activates the transcription of *WEE1* but inhibits p21 expression *via* regulating the REV-ERBs level. Inhibition of BMAL1::CLOCK downregulates *WEE1* leading to mitotic catastrophe and apoptosis and elevates p21 level that causes cell cycle arrest. The two mechanisms collaboratively contribute to the pro-proliferative activity of BMAL1::CLOCK in HCC.

The circadian clock and the cell cycle are interconnected biological circuits both frequently dysregulated in cancer (12). Discovering BMAL1 and CLOCK, the forementioned master transcription factors of circadian rhythms, are required for cancer cell proliferation has encouraged experimental and clinical investigations to inform novel anticancer strategies. Downregulating *BMAL1* or *CLOCK* triggers reduced cell proliferation rate in the glioblastoma stem cells (GSCs), hepatocellular carcinoma (HCC) cells, and leukemia stem cells, featured by a symbolic cellular response of cell cycle dysregulation (13–15). *BMAL1* or *CLOCK* inhibition arrests the cell cycle at the S/G_2_ phase, which is likely associated with the increased expression of p21 and reduced level of CYCLIN B1 (14, 16). Meanwhile, *BMAL1/CLOCK* knockdown induces the downregulation of the *WEE1* gene, which encodes a checkpoint kinase essential for cancer cells’ survival and, consequently, leads to enhanced genome instability and apoptosis (14). Genetic studies demonstrated that transcriptional regulation of *WEE1* and *p21* cooperatively contributes to cancer cell proliferation promoted by BMAL1::CLOCK (14). Taken together, cell cycle regulation is an important cellular mechanism used by the circadian clock for tumor growth control (Figure 1).

Genome instability resulting from compromised DNA damage responses is a major hallmark of cancer that contributes to cancer initiation and progression (17). DNA damage response activates checkpoints that enforce cell cycle arrest to allow DNA repair before cell division (18) and therefore shares similar regulation by the clock as in the cell cycle pathways. Besides, studies have found that the core clock components may regulate earlier stages of the DNA damage response *via* interaction with the damaged DNA or molecules involved in DNA damage detection and signaling. For example, the CLOCK protein was reported to relocalize to the DNA lesion sites and participate in the checkpoint activation (19). Consequently, loss of CLOCK or BMAL1 in mice results in enhanced sensitivity to DNA cross-linking agents (20). CRYs are regulated by DNA damage through post-translational modification and subsequently protect genome integrity by modulating ATR activity (21, 22). PERs physically interact with the checkpoint proteins ATM and CHK2, promote DNA damage-induced apoptosis, and protect animals from tumor development (23–25). The clock factor TIMELESS interacts with the DNA damage response kinases CHK1 and ATR and contributes to DNA damage checkpoint response (26). Overall, most circadian clock components are generally positive regulators of the DNA damage response, thereby suppressing cancer development in healthy tissues. Notably, in prostate cancer, the CRY1 expression is elevated by androgen, endowing it with pro-tumorigenic functions to promote DNA repair and cancer cell survival after ionizing radiation treatment (27).

In addition to DNA damage response, the circadian clock machinery strictly controls the activity of DNA damage repair, particularly the nucleotide excision repair (NER) pathway which specifically repairs DNA lesions caused by UV irradiation, cigarette smoke, and the cross-linking agent cisplatin. The underlying mechanisms involve the clock-controlled circadian expression of xeroderma pigmentosum group A (XPA), an essential scaffold protein of the NER machinery, leading to its peak expression at ZT10 (28). Besides, because the NER repair is facilitated by locally activated transcription, DNA damages at rhythmically expressed genes exhibit a circadian repair on the transcribed strand in phase with the rhythmicity of gene expression (29). Another important link between the clock and DNA damage repair is that the circadian-controlled metabolite nicotinamide adenine dinucleotide (NAD+) acts as the substrate for the poly(ADP-Ribose) polymerase 1 (PARP1) and sirtuin enzymes that contribute to genome stability through participation in the base excision repair (BER), homologous recombination (HR), non-homologous end-joining (NHEJ), and NER (30, 31).

## Circadian clock regulation of the classical oncogene *Myc*

The C-MYC proto-oncogene is a multifunctional transcription factor implicated in many aspects of cellular processes, including proliferation, differentiation, metabolism, and apoptosis (32). Multiple lines of evidence indicate that the cellular level of MYC protein is regulated by the circadian clock. For example, in mice, ubiquitin-mediated MYC degradation is enhanced by CRY2, which recruits the SCF^FBXL3^ E3 ligase to the phosphorylation-modified MYC (33). Consequently, the absence of CRY2 leads to MYC upregulation and enhanced incidence of lymphoma (33). On the other hand, in GSCs, BMAL1 directly binds the promoter region of MYC. Disrupting BMAL1/CLOCK or overexpressing CRY1 decreases MYC levels and impairs GSC proliferation (13). In lung cancer, conversely, *Bmal1* works as a tumor suppressor whose loss leads to increased C-MYC expression and enhanced proliferation (34). Taken together, the MYC regulation reflects cancer-specific functions of the core clock components.

## Circadian clock in cancer stem cells

Cancer stem cells (CSCs) are maintained as a subpopulation of tumor cells by self-renewal. Due to this property and their inherent resistance to mainstream treatments, CSCs are key players in tumor relapses (35). As compared to normal neural stem cells, the GSCs are highly sensitive to perturbation of the circadian pathway. For example, the stemness of GSCs was lost upon *BMAL1* or *CLOCK* downregulation, as presented by reduced frequency of sphere formation and decreased expression of core GSC maintenance transcription factors, including SOX2 and OLIG2 (13). Similarly, targeting *Clock* or *Bmal1* also leads to reduced self-renew capacity and differentiation of murine leukemia stem cells (15).

## Circadian clock regulation of cancer metabolism

Chronic jet lag may cause spontaneous development of HCC following global liver metabolic dysfunction and increased incidence of non-alcoholic fatty liver disease (NAFLD) (36). This suggests that metabolic disruption plays an important role in HCC development associated with circadian dysfunction. In addition, BMAL1 and CLOCK maintain GSCs by promoting glycolysis and mitochondrial oxidative phosphorylation (OXPHOS), two major mechanisms of energy production for cancer cells, supported by reduced oxygen consumption rate (OCR) and extracellular acidification rate (ECAR) upon *BMAL1* or *CLOCK* knockdown (13). Chromatin binding of BMAL1 is reprogrammed in GSCs to directly regulate multiple metabolic enzymes involved in glycolysis and the TCA cycle which is tightly coupled with OXPHOS (13). In addition, the molecular clock was suggested to modulate the activity of autophagy that plays a vital role in fueling the metabolic demands of cancer cells (37). Overall, the molecular mechanisms of circadian clock-regulated cancer metabolism involve both anabolism and catabolism.

## Circadian regulation of tumor microenvironment

The molecular clock has been demonstrated to play a role in tumor microenvironment (TME). For example, BMAL1::CLOCK recruits immune-suppressive microglia cells to the TME of glioblastoma (GBM) *via* transcriptional activation of the *OLFML3* gene (38). Circadian expression of the co-stimulatory molecule CD80 in dendritic cells governs a circadian response of tumor antigen-specific CD8+ T cells to melanoma development (39). The CLOCK protein interacts with HIF-1 to activate VEGF expression, facilitating angiogenesis and metastasis in colorectal cancer (40).

## Circadian clock roles in aging

Increasing evidence has linked disruption of the circadian clock function to aging (41). During the aging process, the circadian control declines, resulting in dampened and occasionally shifted oscillations of sleep-week cycle, body temperature, suprachiasmatic nucleus (SCN) activity, hormone release, and plasma glucose levels (41–43). Mice deficient in BMAL1, CLOCK, or PER show reduced life span and premature aging phenotypes, including sarcopenia, osteoporosis, loss of soft tissues, and cataracts (44–47).

Circadian alignment of feeding time combined with >12 h fasting interval (time-restricted feeding, TRF) enhances the lifespan benefits of calorie restriction (CR), indicating that optimizing the phase of circadian gene expression may be a powerful intervention for increasing life span and wellbeing (48). In addition to optimizing the expression of catabolic factors and disease markers, long-term CR and TRF enhance the circadian amplitude of the core clock and output genes (49, 50) and thereby entrain the clock in the SCN or peripheral organs, respectively (51). Interestingly, in both flies and mice, the lifespan extension benefits of CR require core clock genes (52–54). CR induces globally reprogrammed protein acetylation whose cycling organizes the enhancement of circadian oscillations (55). Therefore, reprogramming circadian rhythms holds significant importance for increased longevity achieved by feeding regimens.

Providing molecular mechanisms, deficiency of the *BMAL1* gene accelerates aging in both human and cynomolgus monkey mesenchymal progenitor cells (MPCs), attributable to a non-canonical transcription-independent role of BMAL1 in stabilizing heterochromatin which prevents activation of pro-senescence retrotransposons (56). Furthermore, comparative transcriptomic analysis showed that a species’ maximum lifespan negatively correlates with the expression of genes responsible for cellular energy metabolism which are more prone under circadian regulation potentially to avoid persistent high expression (57).

The NCI Annual Plan and Budget Proposal for Fiscal Year 2020 stated that the “greatest risk factor for cancer is advancing age”. The fundamental roles of the clock in aging regulation indicates that anti-aging may be an important mechanism used by the circadian clock in cancer prevention. This concept encourages a healthy circadian rhythm reinforced by chrono-nutrition in everyday practice aiming to combat age-related diseases including cancer.

## Circadian disruption in cancer

Transcriptome analysis of patient specimens revealed that a large body of genes cycling in non-cancerous tissues, including core clock genes, may lose oscillations in tumors. For example, serum shock stimulated clear circadian rhythms in the non-tumorigenic breast epithelial cells MCF10A, while the oscillation patterns were not observed in the breast cancer cells MCF7 (7). The CYCLOPS algorism identified quite a number of transcripts cycling in non-cancerous liver samples, including *CRY1*, *PER1*, and *ARNTL2*, lost rhythmic expression in biopsies of human HCC (9). Notably, circadian transcription and metabolism in the liver could be reprogrammed by distal tumors in the lung or breast, highlighting the effects of the tumor macroenvironment on systemic homeostasis of circadian rhythms (58, 59).

Several possible mechanisms can explain circadian disruption during tumorigenesis. C-MYC is a transcription factor constitutively expressed in over 70% of human cancers (32). It has been proposed to disrupt the circadian rhythms in tumor cells by binding an E-box motif element that drives activation of the core clock gene *NR1D1* (60). In epigenetic studies, we reported that, relative to normal neural stem cells, the genome-wide BMAL1 occupancy was considerably enhanced in GSCs coupled with reprogrammed chromatin structure (13). The ectopic recruitment of BMAL1 to thousands of additional binding sites drives essential hallmarks of cancer progression in these cells, including ramped-up metabolism and proliferative activity and maintenance of CSC state (13). Thus, cancer cells extensively reprogram the circadian rhythms to render novel oncogenic functions supporting disease physiology. Cell-specific chromatin structure guides deposition of the master circadian clock transcription factors and is thus a pivotal physiological mechanism defining heterogenous circadian rhythms in health and disease (61, 62). Notably, cancer reprogramming of the circadian machinery does not necessarily result in disturbance of the relatively robust central clock oscillation (13, 15).

## Chrononutrition and circadian medicine in cancer treatment

As narrated above, maintaining a healthy circadian rhythm through consistent daily sleep patterns and diet increases resilience in fighting cancer. In parallel, efforts have been made to identify chemicals that can strengthen our circadian rhythms aiming to overcome circadian clock dampening associated with aging. For example, the small molecule Nobiletin, a naturally synthesized compound enriched in citrus peels, enhances the circadian clock amplitude and protects against metabolic diseases by agonizing the ROR nuclear receptors (63).

In addition to disease prevention, exciting approaches for the circadian clock management of diseases have been developed to limit eating windows, modify drug scheduling, or target clock components. Independent epidemiological studies discovered that the risk of breast cancer and prostate cancer was significantly associated with late night eating behavior (64–66). In mouse orthotopic models, TRF markedly inhibits tumor initiation, progression, and metastasis of obesity-driven postmenopausal breast cancer accompanied by restored circadian gene expression in the tumor suggesting that TRF might suppress tumorigenesis by regulating tumor clock (67). Similarly, in a transgenic MMTV-PyMT model of spontaneous breast cancer, TRF prevented the procarcinogenic effects of high-fat diet (68). These studies support the beneficial role of chrono-nutrition in cancer management and have encouraged clinical trials to prescribe TRF for the treatment of cancer in humans (ClinicalTrials.gov, identifier NCT05259410).

Clinically actionable genes strongly correlate with clock genes in transcriptional expression, suggesting that circadian drug efficacy should be necessarily investigated and considered (3, 7). Synchronizing drug delivery with a patient’s biological clock, known as chronomedicine, has been clinically validated for the improvement of drug efficacy in pathological conditions such as asthma, hypertension, and cardiovascular disease (69). Extending the boundary of chronotherapy further to the surgical procedures, patients who received cardiac surgery in the afternoon and subsequently suffered from major heart damage were half the patients who underwent the same surgery in the morning (70).

In cancer clinical trials, nevertheless, the application of chronochemotherapy has produced inconsistent results within different clinical trials and patient populations. For example, chronochemotherapy benefits observed in a group of 31 women with ovarian cancer (71) were not replicated in a larger study (72). In a retrospective study reviewing 166 glioblastoma patients, Damato et al. found that patients taking morning temozolomide exhibited longer overall survival compared to evening, especially in MGMT-methylated patients (73). However, when the same group conducted a small feasibility study, they found that among 35 patients on different dosing schedules there was no significant difference in either adverse side effects or survival (74). A phase III trial of 564 colorectal cancer patients from 10 European countries receiving chronomodulated infusion of fluorouracil, leucovorin, and oxaliplatin revealed no benefit of the chronotherapy to the whole study population; however, compared with the conventional treatment group, the risk of death was decreased by 25% for men but increased by 38% for women treated with chronotherapy (75). These confusing clinical trials exemplify the heterogeneity of the circadian rhythms in cancer patients, warranting an aforehand definition of the circadian phase potentially through evaluation of appropriate blood-based biomarkers.

By contrast, the emerging fundamental roles of core clock components in cancer biology have urged developing and examining small molecules directly targeting the clock proteins. Encouragingly, existing clock-targeting small molecules have exhibited specific tumor growth inhibition in glioblastoma and other cancer types (10, 13, 37). Considering the broad roles of the core clock components in managing specific cancers, it is favorable for future endeavors to develop cancer-specific strategies with the goal of improving standard first-line regimens. A better understanding of the clock-cancer interactions will inform blood- or tissue-based biomarkers for molecular stratification of cancer patients that may show differential sensitivity to clock-targeting treatments.

## Conclusion

**FIGURE 2:**
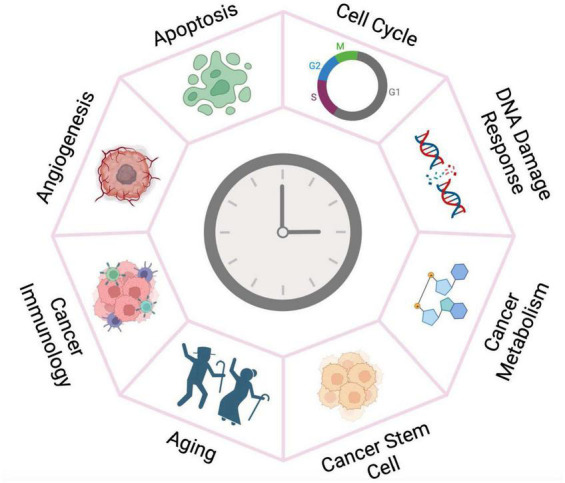
The circadian clock plays versatile roles in cancer development. The circadian clock machinery is reprogrammed by oncogenic pathways and chromatin remodeling in cancer tissues, thereby gaining fundamental functions in cancer development. Targeting the clock may therefore have more deleterious impact on the growth of tumor tissue than for the normal cells.

The molecular clock is emerging as a fundamental regulator of cancer development and prognosis. Here we review recent endeavors that collectively reveal the clock’s multifaceted roles in different aspects of cancer development (Figure 2). Further mechanistic investigation in various cancer systems and pathways assisted by clinical and omics data collected from cancer patients will expand the functional landscape of the circadian clock aiming to guide pharmacological strategies.

## Author contributions

The author confirms being the sole contributor of this work and has approved it for publication.
